# Supplementary material for: Metabolism-Related Gene Expression in Circulating Tumor Cells from Patients with Early Stage Non-Small Cell Lung Cancer
Source: Cancers (Basel). 2022 Jun 30;14(13):3237. doi: 10.3390/cancers14133237 (PMC9264894; doi:10.3390/cancers14133237)
Supplement: Supplementary file 1 [file cancers-14-03237-s001.zip › cancers-1720752-supplementary.pdf]

Table S1. PCR primers and probes sequences.

HK2	**Forward**	5′-CCCTCTACAAGCTACATCCTCACTT-3′	60.6
	**Reverse**	5′-CACGGATGCGGCAGGC-3′	61.8
PHGDH	**Forward**	5′-GGGAGCGGAAGAAGTTCA-3′	55.4
	**Reverse**	5′-CTATAGTCTTCATCCCAAAGGAC-3′	54.8
MCT1	**Forward**	5′-CATGATTGTTGGTGGCTGC-3′	54.9
	**Reverse**	5′-GAAGGCAAGCCCAAGACC-3′	56.5
	**TaqMan Probe**	5′-FAM-TCAGGCTGTGGCTTGATTGCA-BHQ-3′	59.8

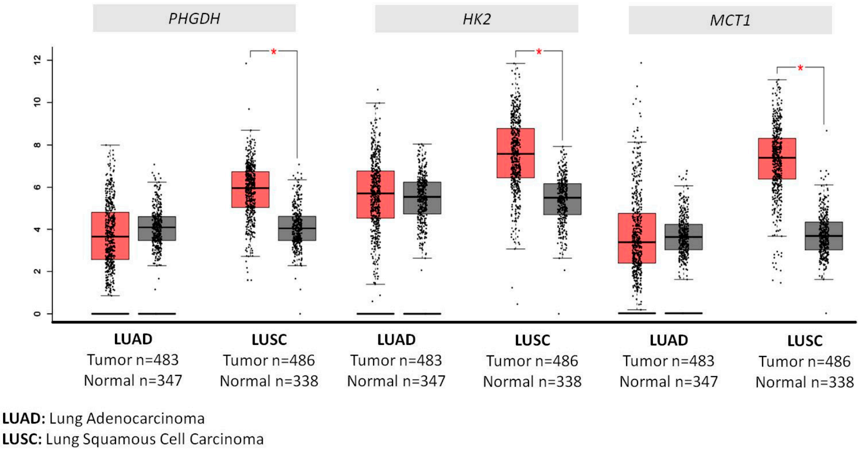

Figure S1. A plot of gene expression level between cancerous and normal tissues based on TCGA analysis.

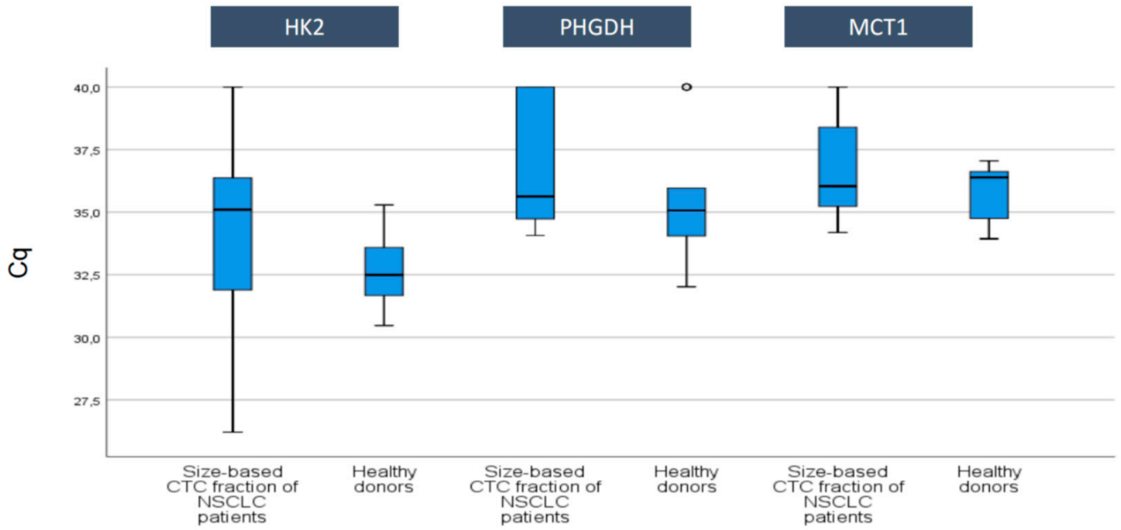

Figure S2. Cq values of each tested gene at baseline in the size-based CTC fractions of early stage NSCLC patients and healthy donors.
